# Supplementary figures and images for: Dopaminergic Modulation of Local Non-oscillatory Activity and Global-Network Properties in Parkinson’s Disease: An EEG Study
Source: Front Aging Neurosci. 2022 Apr 29;14:846017. doi: 10.3389/fnagi.2022.846017 (PMC9106139; doi:10.3389/fnagi.2022.846017)

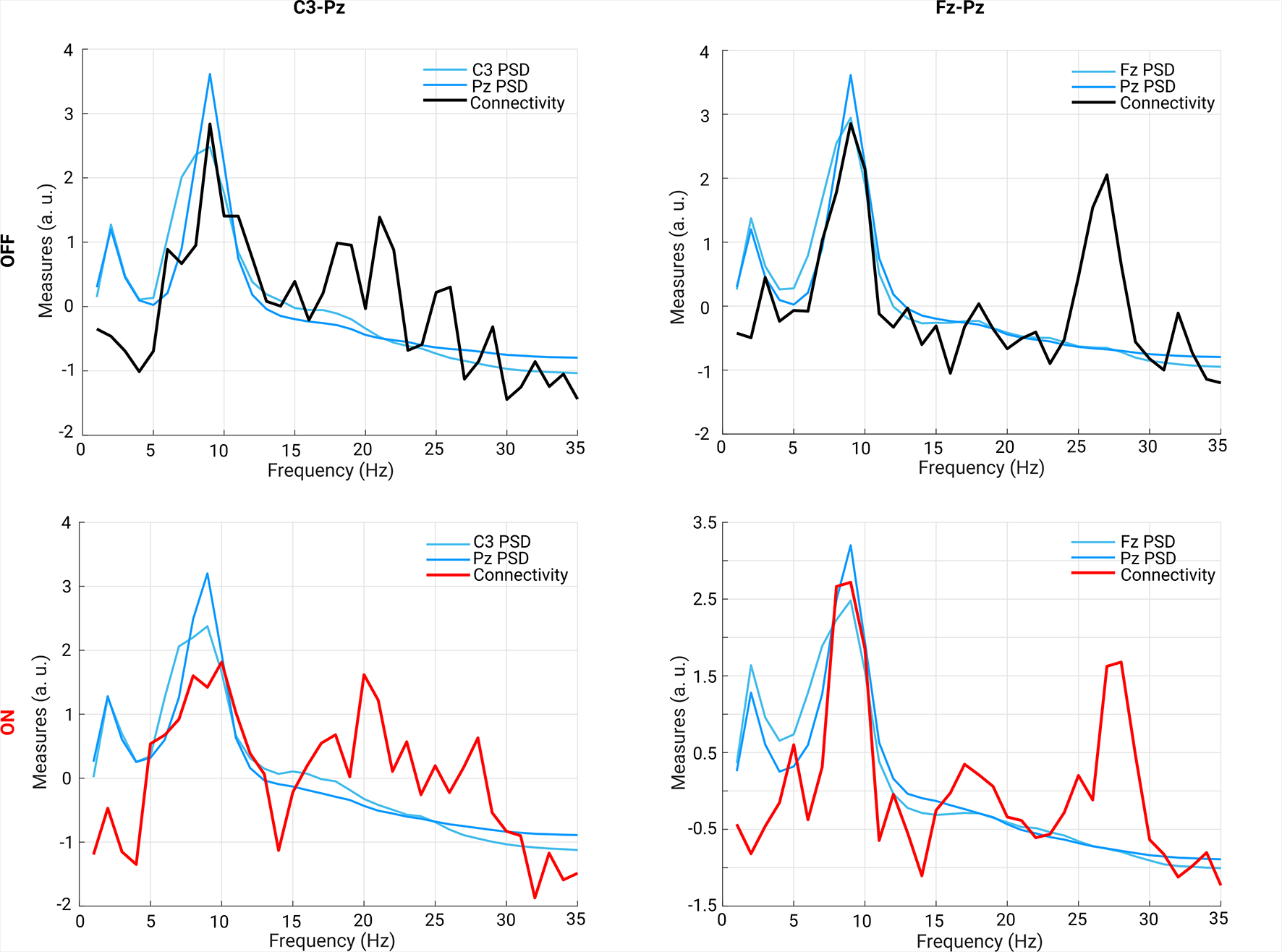

Supplement: Supplementary Figure 1 — Normalized PSDs and connectivity profiles in OFF and ON conditions. In the upper panel: in OFF state, blue lines show the PSD profiles and black lines show the connectivity (left for C3-Pz and right for Fz-Pz). In the lower panel: in ON state, blue lines show the PSD profiles and red lines show the connectivity (left for C3-Pz and right for Fz-Pz). [file Image_1.tiff]

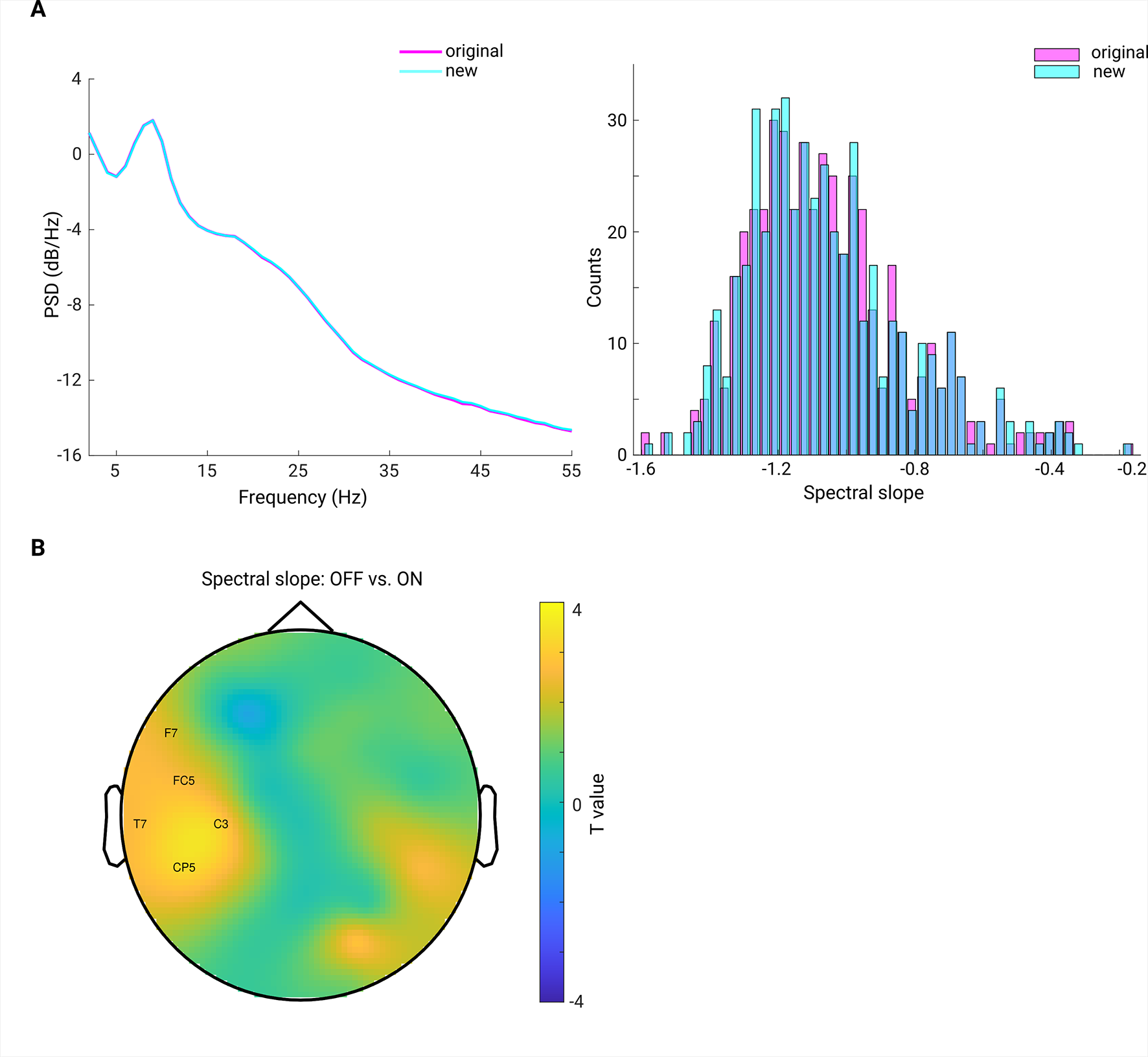

Supplement: Supplementary Figure 2 — Spectral slope effect remains the same after taking care of the cutting borders. (A) Left panel: grand mean PSD plot in PD ON condition before and after respecting the cutting borders (original PSD estimation in magenta and PSD estimation with taking care of the cutting borders in light blue). Two lines almost completely overlap across all the frequencies. Right panel: histograms of the estimated spectral slope values across all the channels and all the subjects within PD ON group with original approach in magenta and new approach (considering the borders) in light blue. (B) Topographical pattern of the comparison of the spectral slope between two conditions based on the estimations considering the cutting borders (OFF vs. ON, p = 0.0240). This topography is consistent with the Figure 2C of the main manuscript. [file Image_2.tiff]

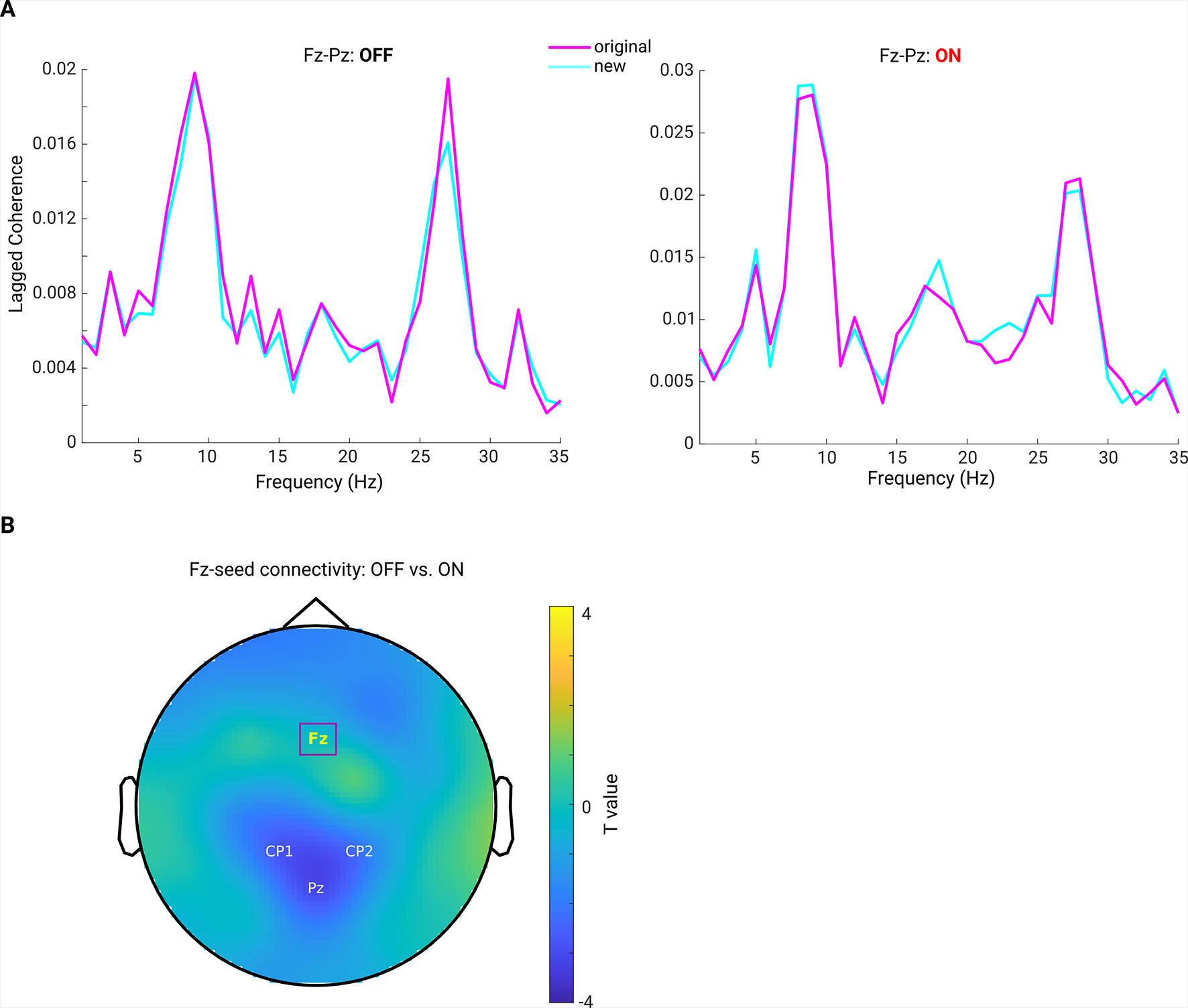

Supplement: Supplementary Figure 3 — Functional connectivity effects remain unchanged after taking care of the borders introduced by removing the artifactual segments. (A) Left panel: averaged functional connectivity between Fz-Pz channels in PD OFF condition before and after considering the cutting borders (original and new estimation in magenta and light blue color, respectively). Two approaches give rise to very similar estimation values. Right panel: same analysis but in the PD ON condition. (B) Topographical pattern of the comparison of the Fz-seed based functional connectivity between two conditions (OFF vs. ON, p = 0.028). The significant cluster is highlighted by the labels in white, while the seed channel Fz is marked in yellow outlined by a square box. This spatial difference pattern is very consistent with the lower panel of Figure 4B of the main manuscript. [file Image_3.tiff]

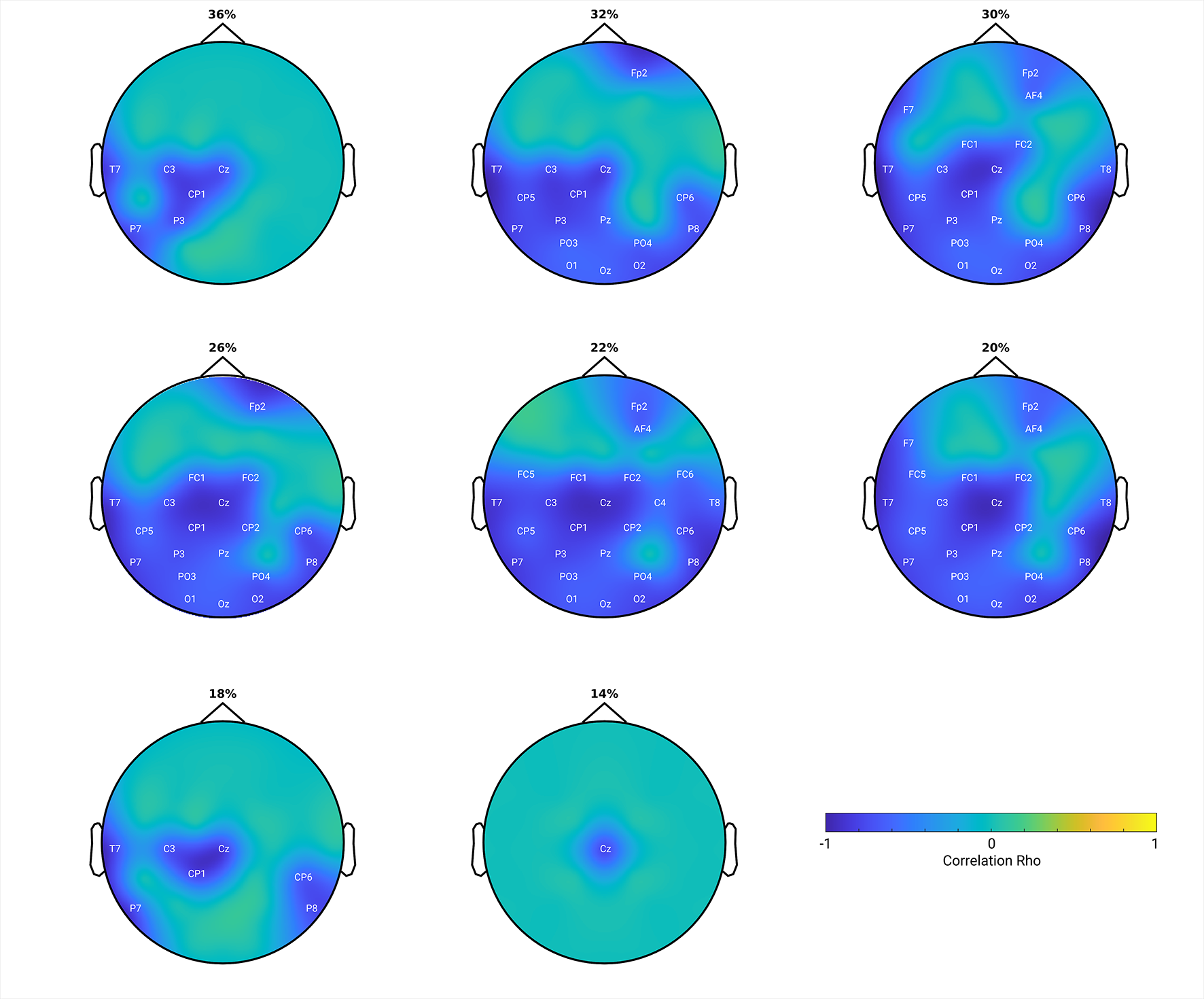

Supplement: Supplementary Figure 4 — Spatial patterns for the correlation between local slope and global efficiency (beta network and under a variety of thresholding values). Significant channels are shown in labels (p < 0.05) after FDR correction. Color bar indicates the correlation coefficient value. The spatial specificity over the centro-parietal region is generally consistent across a family of thresholding values (36–18%). At the PT% of 14%, a significant negative relationship is still present. For the higher PT% values, no significant correlation remains after multiple testing correction. [file Image_4.tiff]
